# Case Report: Selective hepatic resection guided by indocyanine green short-wave infrared fluorescence imaging in a dog with multifocal liver lesions

**DOI:** 10.3389/fvets.2025.1678363

**Published:** 2025-10-10

**Authors:** Soyoung Jang, Yujin Kim, Sunyoung Kim, Sungin Lee

**Affiliations:** ^1^Department of Veterinary Surgery, College of Veterinary Medicine, Chungbuk National University, Cheongju, Republic of Korea; ^2^Department of Veterinary Clinical Sciences, College of Veterinary Medicine, Purdue University, West Lafayette, IN, United States

**Keywords:** indocyanine green, fluorescence imaging, image-guided surgery, hepatic tumors, multiple tumors, canine hepatocellular carcinoma-cholangiocarcinoma

## Abstract

An 11-year-old female spayed Maltese was presented with multiple hepatic masses identified on abdominal ultrasonography and triphasic computed tomography. Lesions were distributed across the left lateral, left medial, quadrate, and caudate liver lobes. A large multicystic mass originating from the left lateral lobe was considered at high risk of rupture, leading to the palliative surgery. Indocyanine green (ICG) was intravenously administered at a dose of 0.5 mg/kg 24 h prior to surgery. During laparotomy, the liver was assessed using a short-wave infrared (SWIR) fluorescence imaging system. Only the mass arising from the left lateral lobe exhibited ICG fluorescence, while no fluorescence was detected in the other lesions. Based on these findings, a left lateral lobectomy including the ICG-positive mass was performed, and punch biopsies were taken from the non-fluorescent lesions in other lobes. Histopathologic examination confirmed the mass from the left lateral lobe as combined hepatocellular carcinoma-cholangiocarcinoma with tumor-free margins. The biopsied lesions were diagnosed as vacuolar hepatopathy. Follow-up evaluation over 6 months revealed no evidence of metastasis. This case demonstrates the potential clinical utility of ICG-SWIR fluorescence imaging in not only detecting and achieving complete resection of tumors, but also supporting real-time intraoperative decision-making, which enables selective resection of malignant tissue while sparing benign lesions.

## 1 Introduction

Hepatic lesions in dogs can be classified into massive, multifocal, and diffuse types based on their distribution pattern. Multifocal hepatic lesions are characterized by the presence of multiple lesions across several hepatic lobes and can arise from various etiologies, including inflammatory changes, benign proliferations, and malignancies (1, 2). Surgical resection, including partial or complete liver lobectomy, is a treatment option for massive liver tumors and generally offers a favorable prognosis if complete excision is achieved (3–5). However, multifocal or diffuse liver tumors involving multiple lobes typically have a poor surgical outcome due to the difficulty in achieving complete resection and the high metastatic potential (3, 4).

In human medicine, several staging systems and surgical algorithms for multifocal hepatic tumors have been developed (6–10). According to the Barcelona Clinic Liver Cancer (BCLC) guideline, surgical resection is recommended for patients with a single tumor or up to three small tumors, while liver transplantation, locoregional therapies, or systemic treatments are favored for those with multifocal lesions involving different lobes (7). However, recent studies have demonstrated that some patients with multifocal hepatic tumors in clinical practice may benefit from hepatectomy, prompting discussion around expanding surgical indications beyond those recommended by the BCLC guideline (6, 8–10). In veterinary medicine, there is no standardized staging system or treatment guidelines for multifocal hepatic tumors, and treatment decisions are often individualized based on each patient’s condition. This underscores the need for systematic studies to establish objective treatment criteria and evidence-based surgical strategies for multifocal hepatic tumors in veterinary patients.

Indocyanine green (ICG) is the first fluorescent dye approved by the U.S. Food and Drug Administration, emitting light at wavelengths approximately 830 nm when excited at 750–800 nm (11–13). Following intravenous (IV) injection, ICG rapidly binds to plasma proteins without altering their structure, and is therefore considered non-toxic (11, 14). Indocyanine green is selectively absorbed by hepatocytes and excreted exclusively into the bile without metabolism (11, 15). In Ishizawa et al. (16) reported the intraoperative use of ICG fluorescence imaging for detecting liver tumors and highlighted differences in biliary excretion between normal hepatocytes and malignant tissues. Malignant tissues are unable to excrete bile normally, leading to intracellular accumulation of ICG and resulting in fluorescent signal emission (5, 15, 16).

In veterinary surgery, ICG fluorescence imaging has been used intraoperatively to aid in tumor detection and to ensure complete resection (5, 15). However, there have been no reports where surgical decisions regarding tumor resection were made intraoperatively based on the presence or absence of ICG fluorescence. This case report describes the application of the ICG-SWIR imaging system to assist intraoperative surgical decision-making in a dog with multifocal hepatic lesions, ultimately enabling the selective resection of a malignant tumor.

## 2 Case description

### 2.1 Case description and preoperative findings

An 11-year-old female spayed Maltese weighing 3.84 kg was referred for evaluation of an intra-abdominal mass identified during a health examination at a local veterinary hospital. Physical examination revealed no abnormalities. A complete blood count showed thrombocytosis (673,000/μL; reference range [Ref]: 148,000–484,000) and reticulocytosis (140,000/μL; Ref: 10,000–110,000). Serum chemistry showed elevated aspartate aminotransferase (76 IU/L; Ref: 23–66), alanine aminotransferase (221 IU/L; Ref: 21–102), gamma-glutamyl transferase (12 IU/L; Ref: 1–10), and alkaline phosphatase (1,242 IU/L; Ref: 29–97). All other blood test results were within normal limits.

**Figure 1:**
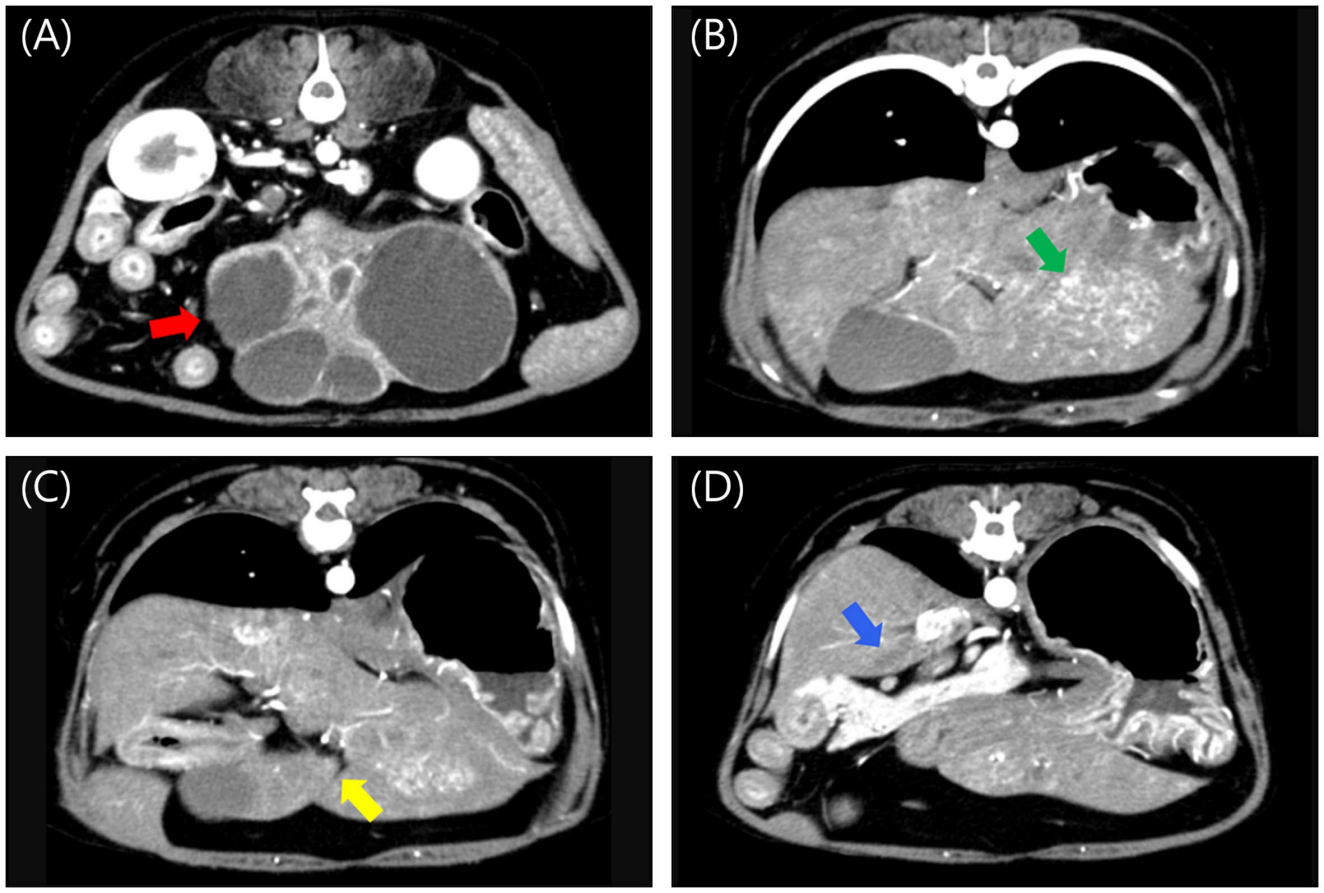
Post-contrast arterial phase computed tomography images of multiple liver masses. **(A)** A large, amorphous, pedunculated, contrast-enhancing mass (#1, red arrow) with internal cavitations originating from the left lateral lobe, measuring 7.29 × 4.37 × 4.06 cm (W × H × L). **(B)** A heterogeneous, contrast-enhancing mass (#2, green arrow) in the left medial lobe, measuring 3.12 × 2.02 × 2.97 cm (W × H × L). **(C)** An isoattenuating, contrast-enhancing mass (#3, yellow arrow) in the quadrate lobe, measuring 0.84 × 0.71 × 0.73 cm (W × H × L). **(D)** An isoattenuating, non-enhancing mass (#4, blue arrow) in the caudate process of the caudate lobe, measuring 0.92 × 0.36 × 0.66 cm (W × H × L). W, width; H, height; L, Length.

Abdominal radiography revealed a relatively well-defined soft tissue opacity mass with a round-to-amorphous shape in the mid-abdomen. Abdominal ultrasound identified a multicystic, amorphous-shaped mass measuring approximately 7 cm in diameter, most likely arising from the left lateral hepatic lobe. A heterogenous, round-shaped mass approximately 2.5 cm in diameter with significant blood flow was also detected, likely in the left medial lobe. Triphasic computed tomography (CT) imaging (Hi Speed QX/I, GE Medical Co., Milwaukee, WI, USA), which had been performed at a local hospital prior to referral, identified multiple hepatic masses. A heterogeneous, amorphous, pedunculated mass (#1) originating from the left lateral lobe measured 7.29 × 4.37 × 4.06 cm (W × H × L) and exhibited contrast enhancement in the arterial, portal, and delayed phases, with rapid washout observed in the delayed phase. The mass contained at least six well-defined non-enhancing cystic lesions. A heterogeneous mass (#2) in the left medial lobe measuring 3.12 × 2.02 × 2.97 cm (W × H × L) was contrast-enhancing in the arterial and portal phases but non-enhancing in the delayed phase. An ill-defined, non-enhancing nodule measuring approximately 2 mm was also identified in the left lateral lobe. A mass (#3) in the quadrate lobe measuring 0.84 × 0.71 × 0.73 cm (W × H × L) was isoattenuating and demonstrated contrast enhancement in the arterial and portal phases but not in the delayed phase. A mass (#4) in the caudate process of the caudate lobe measuring 0.92 × 0.36 × 0.66 cm (W × H × L) was isoattenuating and non-enhancing in all phases (Figure 1).

The largest mass (#1), which was pedunculated from the left lateral lobe and had a cystic internal structure, posed a high risk of rupture due to its size and structure. Palliative surgery was planned to remove it, without performing fine-needle aspiration (FNA) or biopsy. The lesion in the left medial lobe was relatively larger than the others, prompting consideration of complete resection of the left hepatic division. Because surgery was already planned and biopsy is considered more sensitive than FNA among the diagnostic techniques for hepatic lesions in dogs (17), FNA was not performed for the other lesions.

### 2.2 Preoperative management and anesthesia

Indocyanine green (25 mg; Cellbiongreen inj., Cellbion, Seoul, Korea) was dissolved in 5 mL of sterile water (5 mg/mL), then diluted 1:10 with sterile saline to prepare a working solution (0.5 mg/mL). The ICG solution was intravenously injected as a bolus at a dose of 0.5 mg/kg, 24 h preoperatively. Vital signs were carefully monitored, along with any cutaneous signs suggestive of an allergic reaction. No adverse events were noted over 24 h, and surgery was subsequently performed.

Premedication was administered with midazolam (0.2 mg/kg IV; Bukwang midazolam inj., Bukwang Pharm, Seoul, Korea), followed by induction with propofol (4 mg/kg IV; Freepol-MCT Inj., Daewon Pharm, Seoul, Korea) and maintenance of anesthesia using 1.3% isoflurane inhalation. Plasmalyte A solution (20–40 mL/h) and remifentanil (0–3 μg/kg/h; Tivare Inj., BC World Pharm, Gyeonggi-do, Korea) were administered by constant rate infusion (CRI) throughout the surgery.

### 2.3 Surgical procedure

The dog was placed in dorsal recumbency and stabilized using a vacuum positioning mat. A midline skin incision was made using a #10 scalpel blade, starting at the xiphoid process and extending caudally toward the pubis.

**Figure 2:**
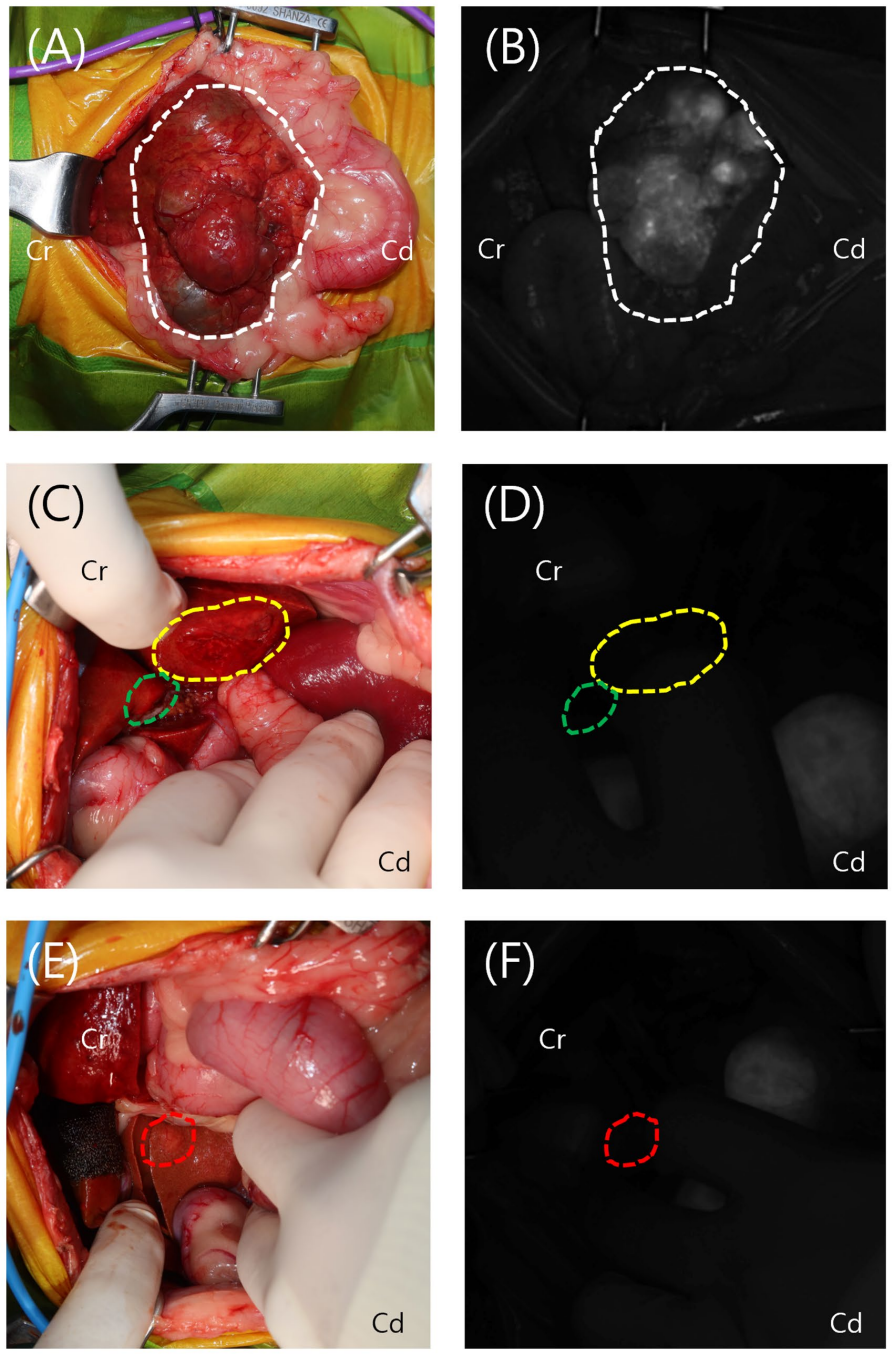
Intraoperative view and visualization of the lesions using the short-wave infrared (SWIR) fluorescence imaging system. **(A,B)** The mass from the left lateral lobe (white dotted line) was grossly identified and exhibited increased indocyanine green (ICG) fluorescence on the SWIR imaging system, displaying a partial fluorescence pattern. **(C–F)** The lesions in the left medial lobe (yellow dotted line), quadrate lobe (green dotted line), and caudate process of the caudate lobe (red dotted line) were grossly identified but showed no ICG fluorescence on the SWIR imaging system.

Upon laparotomy, the mass originating from the left lateral lobe was immediately identified on gross inspection. After ensuring adequate exposure, a short-wave infrared (SWIR) fluorescence-guided imaging system (ZNI; Metaple Bio Co., Ltd., Seoul, Korea) was performed. The mass exhibited increased ICG fluorescence with a partial fluorescence pattern. Adhesions around the mass and the left lateral lobe were carefully dissected using sterile cotton swabs and monopolar electrocautery (Covidien, Mansfield, MA, USA). The entire left lateral lobe, including the mass, was ligated and resected using a vascular/medium, 45 mm, Tri-Staple EndoGIA stapler (Covidien/Medtronic, Dublin, Ireland). In the left medial lobe, quadrate lobe, and caudate process of the caudate lobe, lesions were identified grossly, but no ICG fluorescence was detected on the SWIR imaging system. A 6-mm punch biopsy was performed in each of these areas, followed by application of a gelatin sponge (Spongostan; Ethicon, a Johnson & Johnson company, NJ, USA) and oxidized regenerated cellulose (Surgicel; Ethicon, a Johnson & Johnson company, NJ, USA) for hemostasis (Figure 2). All other liver lobes were visually inspected, palpated, and evaluated using ICG fluorescence to check for additional lesions, and no abnormalities were found. After confirming the absence of intra-abdominal bleeding, abdominal closure was performed.

Postoperative management included a CRI of Plasmalyte A solution combined with vitamin B and taurine (0.5–1.5 mL/kg/h CRI) for fluid and metabolic support, along with remifentanil (5–10 μg/kg/h CRI) for analgesia during the first 24 h. A fentanyl patch (12 μg/h; Durogesic D-trans Patch, Janssen Korea, Seoul, Korea) was applied on postoperative day 2 to maintain analgesia as remifentanil was gradually tapered and discontinued by postoperative day 3. Additional medications included famotidine (1 mg/kg IV, every 12 h; Gaster inj., Dong-A Pharm, Seoul, Korea), cefazolin (22 mg/kg IV, every 12 h; Cephazolin sodium, Chong Kun Dang Pharmaceutical Corporation., Seoul, Korea), vitamin K (1 mg/kg subcutaneously, every 24 h; Vitami-K1 Inj., Dai Han Pharm, Seoul, Korea), and N-acetylcysteine (70 mg/kg IV, every 12 h; Muteran Inj., Han Wha Pharma, Gangwon-do, Korea), all administered for 2 days postoperatively. In consideration of the risk of hypoglycemic shock associated with impaired hepatic function and hepatic lobectomy, a continuous glucose monitoring system (Libre; Abbott, Chicago, IL, USA) was applied. Blood glucose levels were monitored every 2 h postoperatively and ranged from 80 to 200 mg/dL, with no clinically significant abnormalities observed. The patient was discharged 3 days postoperatively. Ursodeoxycholic acid (UDCA; 10 mg/kg orally, every 12 h; Ursa Tab., Dae Woong, Seoul, Korea) and S-adenosyl methionine (SAMe)/silybin (100 mg per dog PO every 24 h; Zentonil Advanced, Vetoquinol, Lavaltrie, QC, Canada) were prescribed for 4 weeks.

### 2.4 Histopathological findings and follow-up

**Figure 3:**
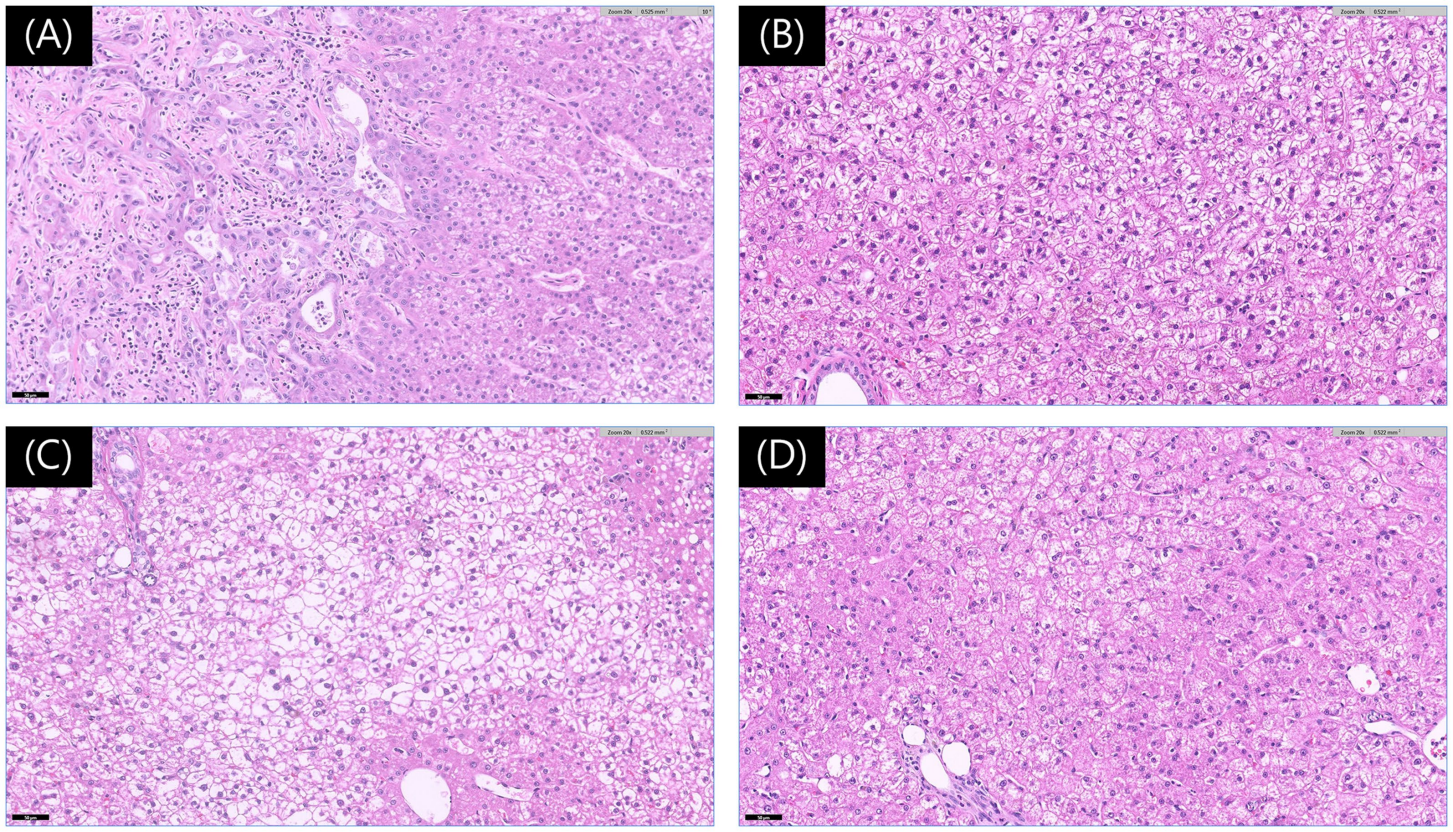
Histopathological findings of liver lesions. **(A)** Photomicrograph of the mass from the left lateral lobe showing features consistent with a hepatocellular-cholangiocellular carcinoma. The image highlights the junctional area with cholangiocellular differentiation (left) and hepatocellular differentiation (right). The neoplastic cells exhibit mild to moderate anisocytosis and anisokaryosis, with prominent nucleoli (hematoxylin and eosin [H&E] stain, Magnification ×200, Scale bar 50 μm). **(B–D)** Photomicrographs of the lesions from the left medial **(B)**, quadrate **(C)**, and caudate **(D)** lobes exhibit features consistent with vacuolar hepatopathy (H&E stain, Magnification ×200, Scale bar 50 μm).

The resected left lateral lobe with the mass, along with the biopsy samples, was preserved in 10% neutral buffered formalin and submitted for histopathologic evaluation (IDEXX Laboratories, Inc., USA). Pathological analysis of the mass revealed a combined hepatocellular carcinoma-cholangiocarcinoma (cHCC-CCA), with a mitotic count of 0 per 10 high-power fields. The surgical margin was tumor-free, exceeding 15 mm, and no vascular invasion was observed. Histopathologic evaluation of the three biopsy specimens revealed vacuolar hepatopathy characterized by combined glycogen and lipid accumulation, which is considered a benign lesion (Figure 3).

On postoperative day 20, clinical evaluation showed no significant abnormalities. The surgical site had healed completely, allowing for suture removal. At 6 weeks postoperatively, liver enzymes had further decreased compared with the immediate postoperative values, indicating continued improvement in the biochemical profile. Aspartate aminotransferase decreased from 161 to 38 IU/L (Ref: 23–66), alanine aminotransferase from 312 to 133 IU/L (Ref: 21–102), gamma-glutamyl transferase from 22 to 15 IU/L (Ref: 1–10), and alkaline phosphatase from 2,345 to 974 IU/L (Ref: 29–97). Follow-up thoracic and abdominal radiographs revealed no signs of metastasis, and generalized hypertrophy of the right liver lobes was noted. Abdominal ultrasonography revealed multiple hyperechoic nodules (approximately 3 mm in diameter) throughout the right liver lobes, and a single hypoechoic, oval-shaped nodule (approximately 2 cm) in the left medial lobe. These lesions had not been detected preoperatively. At 6 months postoperatively, in addition to the previously identified lesions, a 1.6 × 1.7 cm nodule was observed in the right lateral lobe, along with multiple hyperechoic nodules diffusely distributed across all hepatic lobes on ultrasound. FNA of the lesion in the right lateral lobe was performed and revealed vacuolar hepatopathy. As the FNA results indicated a non-neoplastic lesion, the hepatic nodules were considered more likely benign than metastatic. Nonetheless, because metastasis could not be definitively ruled out, continued regular follow-up was deemed necessary.

## 3 Discussion

Real-time fluorescence imaging offers intraoperative guidance and greater sensitivity for tumor detection than visual inspection or palpation (13, 18). Notably, ICG-SWIR fluorescence imaging aids not only in tumor identification but also in surgical planning and achieving tumor-free margins (19, 20). During laparoscopic surgery, the limited surgical field and inability to palpate organs make the SWIR imaging system an essential modality for complete tumor resection (16). In human medicine, SWIR imaging with ICG has been widely used for intraoperative tumor detection and margin determination in patients with various tumors (11, 19–24), and has similarly been applied in veterinary patients (5, 12, 14, 15, 18, 25–27). In this case, ICG-SWIR fluorescence-guided resection enabled complete removal of the malignant lesion with tumor-free margins.

Preoperative imaging and intraoperative visualization revealed multiple hepatic lesions in this patient, but ICG fluorescence was detected only in the mass originating from the left lateral lobe (28). Histopathology confirmed that the fluorescence-positive lesion was malignant, whereas the non-fluorescent lesions were benign, further supporting the utility of intraoperative ICG imaging in surgical decision-making. Recent studies have suggested the potential of ICG fluorescence to differentiate benign from malignant lesions (15, 20, 28). In human medicine, ICG fluorescence showed a sensitivity of 88% and a positive predictive value (PPV) of 77% for distinguishing malignancies from benign or inflammatory lesions (20). In veterinary medicine, a small-scale study reported a sensitivity of 71% and a PPV of 80% for differentiating HCC from nodular hyperplasia in dogs (15). In addition, findings for nodular hyperplasia lesions have been inconsistent, with fluorescence observed in 11 of 12 lesions in one study (5), but in only 1 of 6 lesions in another (15), indicating the need for additional investigation. In this case, the non-fluorescent lesions were diagnosed as vacuolar hepatopathy. To our knowledge, ICG fluorescence in vacuolar hepatopathy has not previously been evaluated, and thus the reason for the absence of fluorescence remains unclear. Given that vacuolar hepatopathy is reported not to involve alterations of the intracellular bile canaliculi (29), we suggest that bile excretion may be preserved, which in turn could explain the absence of ICG accumulation, unlike in neoplastic hepatocytes. However, further studies are needed to determine the exact underlying mechanism. Despite these considerations, this case demonstrates that ICG-SWIR fluorescence imaging provides real-time surgical guidance for determining the extent of resection in patients with multiple hepatic lesions. This approach allows for the selective removal of malignant tissue while preserving normal liver parenchyma. Minimizing hepatic resection helps preserve liver function and reduces the risk of complications due to insufficient residual liver volume (30).

cHCC-CCA is a rare primary hepatic neoplasm in dogs, accounting for approximately 2% of all primary hepatobiliary tumors (31). Histopathologically, the tumor exhibits features of both HCC and CCA (31, 32). In dogs, the median survival time (MST) after surgical resection ranges from 695 to over 1,460 days for HCC, and is less than 6 months for CCA (31, 33). Interestingly, dogs diagnosed with cHCC-CCA have an MST of approximately 700 days, comparable to that of HCC and notably longer than that of CCA (31). These findings suggest that surgical resection, whether partial or complete liver lobectomy, can be a viable treatment option for cHCC-CCA in dogs (3, 34). When multiple lesions are identified intraoperatively in dogs with cHCC-CCA, an aggressive surgical approach may be warranted to prevent postoperative metastasis (31). However, extensive liver lobectomy was not feasible in this case because of masses in multiple lobes. As demonstrated in this report, the SWIR imaging system with ICG may offer real-time intraoperative guidance, supporting selective resection of suspicious lesions. Furthermore, a recent veterinary study on ICG fluorescence patterns in canine hepatic tumors reported that cHCC-CCA exhibited a characteristic partial fluorescence pattern (5). A similar pattern was observed in the present case. Although fluorescence patterns alone may not be sufficient for definitive diagnosis, they aided surgeon to differentiate neoplasms in this report and warrant further investigation.

In conclusion, the SWIR imaging system with ICG was effective in facilitating the selective resection of malignant hepatic tumors in a dog with multifocal liver lesions in this case. When multifocal liver lesions are present and immediate surgical intervention is required due to limitations in preoperative diagnosis or the patient’s condition, this imaging modality may serve as a valuable tool for real-time intraoperative decision-making. Further studies are required to validate the diagnostic accuracy of ICG fluorescence imaging in differentiating malignant from benign lesions and in identifying tumor subtypes in veterinary hepatic oncology.

## Data Availability

The original contributions presented in the study are included in the article/supplementary material, further inquiries can be directed to the corresponding author.
